# Automated mitral valve assessment for transcatheter mitral valve replacement planning

**DOI:** 10.3389/fbioe.2022.1033713

**Published:** 2022-11-16

**Authors:** Patricia Lopes, Paul L. Van Herck, Joris F. Ooms, Nicolas M. Van Mieghem, Roel Wirix-Speetjens, Jan Sijbers, Jos Vander Sloten, Johan Bosmans

**Affiliations:** ^1^ Materialise N.V, Medical Department, Leuven, Belgium; ^2^ Division of Biomechanics—BMe, Department of Mechanical Engineering, KU Leuven, Leuven, Belgium; ^3^ Department of Cardiology, University Hospital Antwerp, Antwerp, Belgium; ^4^ Department of Cardiology, Erasmus M.C, Rotterdam, Netherlands; ^5^ imec-VisionLab, Physics Department, University of Antwerp, Antwerp, Belgium

**Keywords:** pre-interventional planning, automated mitral valve assessment, saddle- and D-shaped mitral annulus, statistical shape model (SSM), transcatheter mitral valve replacement (TMVR), cardiac CT

## Abstract

Transcatheter mitral valve replacement (TMVR) has emerged as a minimally invasive alternative for treating patients suffering from mitral valve disease. The number of TMVR procedures is expected to rise as devices currently in clinical trials obtain approval for commercialization. Automating the planning of such interventions becomes, therefore, more relevant in an attempt to decrease inter-subject discrepancies and time spent in patient assessment. This study evaluates the performance of an automated method for detection of anatomical landmarks and generation of relevant measurements for device selection and positioning. Cardiac CT scans of 70 patients were collected retrospectively. Fifty scans were used to generate a statistical shape model (SSM) of the left heart chambers at ten different timepoints, whereas the remaining 20 scans were used for validation of the automated method. The clinical measurements resulting from the anatomical landmarks generated automatically were compared against the measurements obtained through the manual indication of the corresponding landmarks by three observers, during systole and diastole. The automatically generated measurements were in close agreement with the user-driven analysis, with intraclass correlation coefficients (ICC) consistently lower for the saddle-shaped (ICC_Area_ = 0.90, ICC_Perimeter 2D_ = 0.95, ICC_Perimeter 3D_ = 0.93, ICC_AP-Diameter_ = 0.71, ICC_ML-Diameter_ = 0.90) compared to the D-shaped annulus (ICC_Area_ = 0.94, ICC_Perimeter 2D_ = 0.96, ICC_Perimeter 3D_ = 0.96, ICC_AP-Diameter_ = 0.95, ICC_ML-Diameter_ = 0.92). The larger differences observed for the saddle shape suggest that the main discrepancies occur in the aorto-mitral curtain. This is supported by the fact that statistically significant differences are observed between the two annulus configurations for area (*p* < 0.001), 3D perimeter (*p* = 0.009) and AP diameter (*p* < 0.001), whereas errors for 2D perimeter and ML diameter remained almost constant. The mitral valve center deviated in average 2.5 mm from the user-driven position, a value comparable to the inter-observer variability. The present study suggests that accurate mitral valve assessment can be achieved with a fully automated method, what could result in more consistent and shorter pre-interventional planning of TMVR procedures.

## 1 Introduction

Transcatheter mitral valve replacement (TMVR) therapies have been adopted as a reliable option in the treatment of mitral regurgitation (MR), in an attempt to address the high risk posed by surgical treatment and the limitations of edge-to-edge transcatheter mitral valve repair (Alperi et al., 2021; Aoun et al., 2021). Due to the minimally invasive nature of these interventions, pre-procedural imaging plays a crucial role in guaranteeing successful outcomes (Natarajan et al., 2016). Upon assessment of the valve pathology and severity of valve dysfunction, including the morphologic and anatomic characterization of mitral leaflets (Mackensen et al., 2018), the assessment of the mitral annulus is a pivotal step in TMVR planning. When considering device sizing, excessive oversizing may result in annular rupture or left ventricular outflow tract (LVOT) obstruction. Reversely, insufficient oversizing may result in paravalvular regurgitation or prosthesis embolization (Thériault-Lauzier et al., 2016; Murphy et al., 2017). The non-planar saddle shape of the mitral annulus, in combination with the dynamic changes that it undergoes during the cardiac cycle, represent, however, undeniable challenges in device size estimation.

**FIGURE 1:**
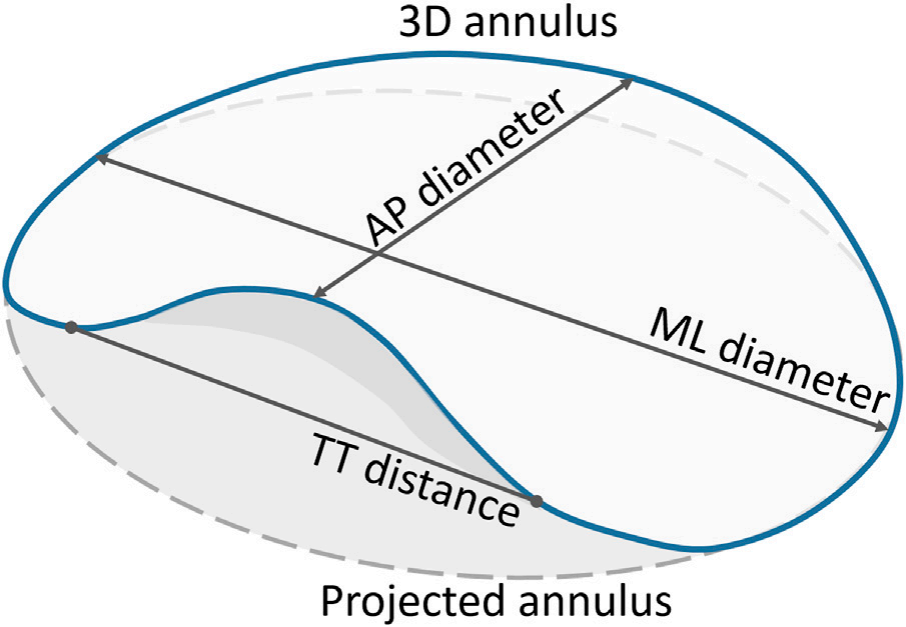
Quantification parameters of the mitral annulus. The full blue curve represents the 3D saddle-shaped annulus, whereas the grey dashed curve represents the projection of the mitral annulus onto its best-fit plane. Both the 3D and the projected 2D perimeters were measured. Only the grey area, corresponding to the projected curve, was calculated. The two main diameters, namely the AP and ML diameters, were measured based on the 3D annulus curve. The TT distance was measured in 3D, with the TT-line replacing the anterior portion of the saddle-shaped curve to represent the D-shaped annulus.

The mitral annulus’ shape approximates a hyperbolic paraboloid, with peaks located anteriorly and posteriorly, and valleys located medially and laterally in close proximity to the fibrous trigones. The anterior horn is anatomically coupled with the aortic valve, in the so-called aorto-mitral continuity, and spans between the two trigones. The posterior annulus encompasses the remainder of the annular perimeter at the insertion of the posterior mitral valve leaflet (Silbiger, 2012; Blanke et al., 2014; Weir-McCall et al., 2018). For the pre-interventional assessment of the mitral valve, the annulus is typically represented as a cubic spline fitted to points placed at regular intervals around the mitral valve center. The annular plane is defined as the least-squares plane fitted to the 3D annular contour. Quantification of the mitral annulus dimensions is performed both in two and three dimensions, with the area commonly calculated based on the 2D spline resulting from the projection of the annular contour onto the annular plane. The maximum and minimum diameters, or alternatively antero-posterior (AP) and medio-lateral (ML) diameters, are evaluated either in 2D or 3D, depending on the study. The annulus perimeter, however, is often evaluated based on both the original 3D and the projected 2D contours (Blanke et al., 2014) (Figure 1).

(Blanke et al., 2014) have proposed the exclusion of the anterior horn of the saddle-shaped annulus for TMVR assessment, creating a D-shaped annulus suitable for planar measurements, with the anterior border a virtual line connecting the fibrous trigones. The main motivation for representing the saddle-shaped mitral annulus as a D-shaped ring is related to the potential mismatch between the device shape and the annular landing zone. Because some devices are cylindrical whereas others are D-shaped, the estimation of the device size needs to take into account the potential obstruction of the LVOT.

Throughout the cardiac cycle, the annulus moves in a passive manner determined by the contraction and relaxation of the adjacent atrial and ventricular walls, and the motion of the aortic root (Silbiger, 2012). Such motion can have a considerable impact in the mitral annulus dimensions across the cardiac cycle, which can determine the success or failure of a device to be implanted through a transcatheter approach.

As the number of procedures is expected to rise, a consistent methodology to characterize the mitral valve and identify the most suitable TMVR device for each patient is of paramount importance. Automated methods can be particularly attractive, as they provide consistent results, while being faster than the standard manual approach. However, the accuracy of such methods needs to be ensured. Therefore, with this study, we aimed at evaluating the performance of a fully automated approach for the assessment of the mitral valve apparatus during pre-interventional planning of TMVR procedures. Specifically, we compare the accuracy of the measurements resulting from this automated method with those resulting from manual indications by three observers, in both diastole and systole.

## 2 Materials and methods

### 2.1 Study population

This retrospective study was approved by the institutional Ethical Committee (Antwerp University Hospital).

The study population consisted of 70 consecutive patients, who underwent a retrospective ECG-gated cardiac CT between November 2017 and January 2019. The CT scan was acquired as a routine diagnostic procedure for the evaluation of thoracic pain. All patients aged 18 or more, with no coronary artery disease and no mitral valve dysfunction were considered eligible for enrolment in the study. Exclusion criteria were insufficient computed tomographic image quality and prior valvular heart intervention.

### 2.2 Imaging protocol

Cardiac CT examinations were performed using a 64-slice GE Lightspeed scanner (GE Healthcare, Milwaukee, WI, United States). Images were obtained with a 64 mm × 0.625 mm slice collimation and a gantry rotation time of 0.35 ms. Tube voltage and current were adapted to patient’s body mass index (100–120 kV; 450–350 mA). A retrospective ECG-triggered scanning protocol was used. For contrast-enhanced image acquisition, a non-ionic contrast agent (Iomeron 350, 110 ml) and saline flush (60 ml) was injected into an antecubital vein using a Nemoto injection system. This injection protocol is triphasic, with 80 ml contrast at a flow rate of 5 ml/s, followed by 30 ml contrast + 30 ml saline at 2.5 ml/s and finally 30 ml saline at 2.5 ml/s.

CT data were subsequently reconstructed at regular locations of the RR-interval, resulting in 10 time points of the cardiac cycle.

### 2.3 Statistical shape model generation

#### 2.3.1 End-diastolic model

The first cardiac phase of each CT scan, corresponding to end-diastole, was segmented in Mimics 21.0 (Materialise N.V., Leuven, Belgium), using a semi-automated left heart segmentation tool. Manual adjustments were performed when deemed necessary, with special attention for the region at the level of the mitral valve. Surface models were then generated from the segmentation masks of the left atrium, left ventricle and aorta. These surface models were subsequently imported in 3-matic 13.0 (Materialise N.V., Leuven, Belgium) for editing, namely by 1) trimming of the pulmonary veins and the ascending aorta, 2) Boolean Union of the three surfaces into a single manifold surface, 3) smoothing, and 4) mesh optimization. Finally, the different surfaces included in the final manifold were labelled using prime numbers between 2 and 19 and the boundaries between surfaces were labelled using the product of the two adjoining surfaces, in a similar manner to that described by (Hoogendoorn, 2013). This resulted in a total of 15 unique labels with values ranging between 2 and 133, for identification of the left heart structures and their boundaries.

Once the surface models of the left heart for the fifty subjects containing analogous surface information were available, a point correspondence method was employed. A template-based method, built upon the works of (Amberg et al., 2007)*,* (Danckaers et al., 2014), and (van Dijck, 2021), was modified to take into account the surface labeling, preventing corresponding points from belonging to different anatomical structures. Finally, a principal component analysis (PCA) was performed to construct the end-diastolic SSM.

#### 2.3.2 Dynamic model

The segmentation of the left heart obtained for the end-diastolic phase was propagated to the subsequent cardiac phases using an automated method available in Mimics 21.0. The end-diastolic SSM was then fitted to the resulting surface models to obtain surface correspondence between the multiple time points of the cardiac cycle and the different subjects. A new SSM was generated including the 500 instances, containing information on both anatomical and dynamic variation between subjects.

The following landmarks were manually indicated on the mean instance of the dynamic SSM: the three aortic valve cusps, namely right coronary, left coronary and non-coronary cusps; the medial and lateral trigones, and the left ventricular apex.

### 2.4 Automated mitral valve assessment

The automated method for assessment of the mitral valve was run on the twenty validation cases, for both the end-diastolic and end-systolic phases, as these correspond to the most relevant phases for TMVR planning. The method was initiated by running a fully automated segmentation method available in Mimics for the separation of the aorta, left ventricle and left atrium, and subsequent generation of the corresponding surface models. Thereafter, the dynamic SSM was aligned with the target case using first a point-set registration based on the centers of mass of the three structures and the centers of the boundaries corresponding to the aortic and mitral valves, followed by an iterative closest point registration. The SSM was subsequently fitted onto the target surfaces using a correspondence-based fitting method as described by (van Dijck, 2021). Finally, the fitted SSM was warped to the target surface for improved matching. The node indices corresponding to landmarks indicated on the mean instance of the SSM are used to extract the coordinates of the landmarks on the target case. Additionally, the mitral and aortic valve annuli are defined by the boundaries of the respective structures (left atrium and left ventricle for the mitral valve, and left ventricle and aorta for the aortic valve).

### 2.5 Manual mitral valve assessment

Three observers, with various backgrounds and experience levels with the image analysis software, performed the manual indication of the mitral annulus landmarks in both the end-diastolic and end-systolic phase. The first observer (PVH) is a cardiologist with more than 10 years of imaging experience and no direct experience with the Mimics software; the second observer (PL) is a biomedical engineer with 7 years of experience in cardiac image analysis, and more than 10 years of advanced use of the Mimics software; and the third observer (JO) is a clinical researcher with 3 years of experience, which included 1 year of regular use of the Mimics software and similar tools.

**FIGURE 2:**
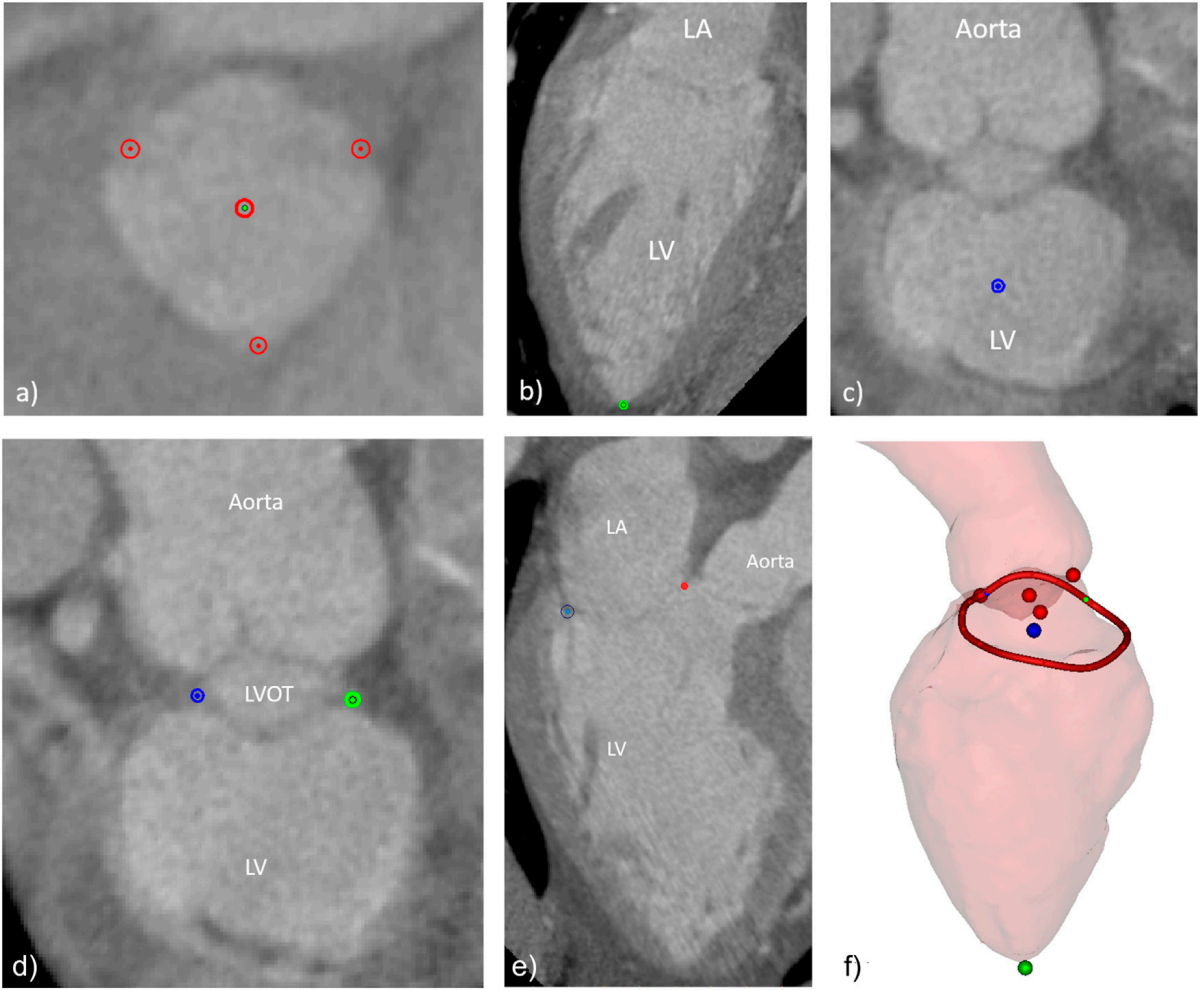
Manually indicated landmarks, including **(A)** aortic valve cusps and center, **(B)** left ventricular apex, **(C)** mitral valve center, **(D)** fibrous trigones, **(E)** mitral annulus points in one of the long axis planes around the mitral valve center, and **(F)** overview of final set of landmarks and the 3D surfaces of the LV and the aortic root (the LA surface was excluded for clarity).

A dedicated workflow was developed, in such way that all users would follow approximately the same procedure, this way avoiding potential methodological discrepancies linked to their experience with the software. The workflow included the indication of the aortic cusps and aortic valve center (Figure 2A), the left ventricular apex (Figure 2B), the mitral valve center (Figure 2C), the medial and lateral trigones (Figure 2D), and the mitral annulus (Figure 2E). All landmarks were visually assessed on the 3D representations of the left heart and adjustments performed when deemed necessary. Figure 2F shows the manually indicated landmarks visualized on the 3D representations of the aorta and left ventricle (the left atrium was excluded for clarity).

### 2.6 Statistical analysis

The ground-truth for each anatomical landmark was defined as the average position of the three points indicated by the observers. For each landmark, the Euclidean distance between the landmark indicated by each of the observers was measured in relation to the average location of the three observers. The inter-observer error was defined as the mean of the three distances. In the case of the automated approach, the inter-method error was defined as the difference between the landmark predicted by the algorithm and the mean landmark location for the three observers. The average landmarks and the measurements were automatically generated using a dedicated software plugin in the Mimics software.

To understand the clinical impact of the errors in the automated approach, a set of relevant measurements to characterize the mitral annulus were estimated. These measurements include 2D and 3D mitral annulus perimeter, the projected mitral annulus area, the antero-posterior (AP) and medio-lateral (ML) diameters, the inter-trigone (TT) distance, the annulus height, and the aorto-mitral angle. For both the manual and automatic approaches, these measurements were generated in a fully automated manner based on the landmarks.

The mean and standard deviation (SD), as well as the median and inter-quartile range were calculated for the measurements performed by the three observers. Furthermore, the inter-observer variability was evaluated by comparing the measurement performed by each observer with the mean measurement of the three observers. Results are presented as mean ± SD. Moreover, the intraclass correlation coefficients (ICC) and 95% confidence intervals were estimated using a single measure, absolute-agreement, two-way random model. Reliability was measured according to the following values of ICC: < 0.5 poor, 0.5–0.75 moderate, 0.75–0.9 good, and >0.9 excellent reliability (Koo and Li, 2016). The inter-method variability, expressed as mean ± SD, was evaluated by comparing the average measurement by the three observers and the automatically generated measurement. The agreement between the manual and the automatic approaches was assessed using single measures, absolute-agreement, two-way mixed effects model. Finally, the agreement between each of the measurements for the manual and automated approaches was investigated through linear regression and Bland-Altman analyses. The statistical analysis was done with Python 3.7, using standard Python libraries (NumPy, SciPy, Pandas).

## 3 Results

### 3.1 Study population

The group of 50 patients used to generate the SSM included 18 males (36%) and had a mean age of 49 ± 10.1 years, whereas 10 of the 20 patients included in the validation group were male (50%) with a mean age of 48 ± 14.8 years.

### 3.2 Statistical shape model

**FIGURE 3:**
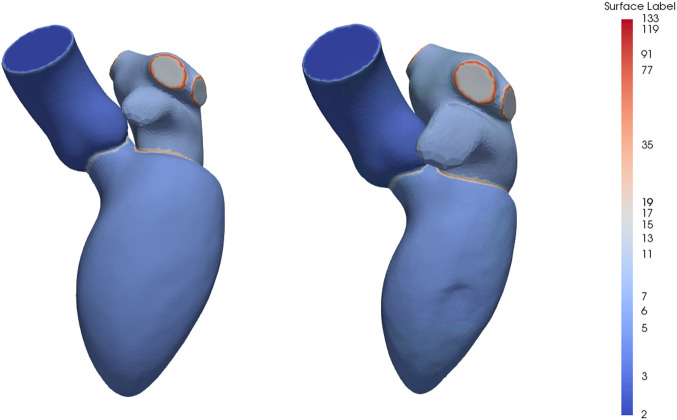
Mean instance of the generated statistical shape models of the left heart chambers for the diastolic phases (left) and full cardiac cycle (right). The labels are prime numbers representing the different structures and the boundaries are obtained by multiplying the labels of the adjoining structures. Specifically, the aorta, left ventricle and left atrium are represented by labels 3, 5 and 7, respectively, resulting in labels 15 for the aortic annulus and label 35 for the mitral annulus.

The mean instance of the generated end-diastolic and dynamic models can be seen in Figure 3. The labels represent the structures and boundaries of the left heart, with labels 3, 5, and 7 for the aorta, left ventricle and left atrium, respectively. Correspondingly, the aortic and mitral annuli are represented by labels 15 and 35.

### 3.3 Manual mitral valve assessment

Table 1 displays the calculated means and standard deviations (SD), as well as medians and interquartile ranges of the mitral annulus measurements resulting from manual indications by the three observers. Statistically significant differences were registered between systolic and diastolic phases for the AP diameter (*p* = 0.0037), the annulus height (*p* = 0.043), and the aorto-mitral angle (*p* < 0.001). When comparing the measurements resulting from the saddle-shaped annulus with the D-shaped curve, all presented significant differences, except for the projected area.

**TABLE 1:** Mitral annulus measurements.

		Saddle-shaped annulus	
Measurement	Units	Diastole	Systole
		Mean ± SD	Mean ± SD
Area 2D	[cm^2^]	9.50 ± 1.8	9.78 ± 1.6
Perimeter 2D	[mm]	116.95 ± 10.4	116.21 ± 8.9
Perimeter 3D	[mm]	123.26 ± 10.9	123.95 ± 9.5
AP Diameter	[mm]	28.90 ± 3.5	30.69 ± 2.7
ML Diameter	[mm]	38.84 ± 3.7	38.13 ± 3.7
Annulus height	[mm]	5.79 ± 1.4	6.65 ± 1.9
Aorto-mitral angle	[°]	127.10 ± 6.7	119.45 ± 8.1
TT-distance	[mm]	23.64 ± 2.4	22.82 ± 2.5

		D-shaped annulus	
Area 2D	[cm^2^]	9.59 ± 1.8	9.77 ± 1.6
Perimeter 2D	[mm]	114.03 ± 10.5	114.25 ± 9.3
Perimeter 3D	[mm]	118.71 ± 10.8	119.23 ± 9.7
AP Diameter	[mm]	28.67 ± 3.1	29.80 ± 2.8
ML Diameter	[mm]	39.29 ± 3.6	38.43 ± 3.6
Annulus height	[mm]	3.62 ± 1.2	4.09 ± 1.5
Aorto-mitral angle	[°]	128.81 ± 7.2	122.07 ± 8.7
TT-distance	[mm]	23.64 ± 2.4	22.82 ± 2.5

**FIGURE 4:**
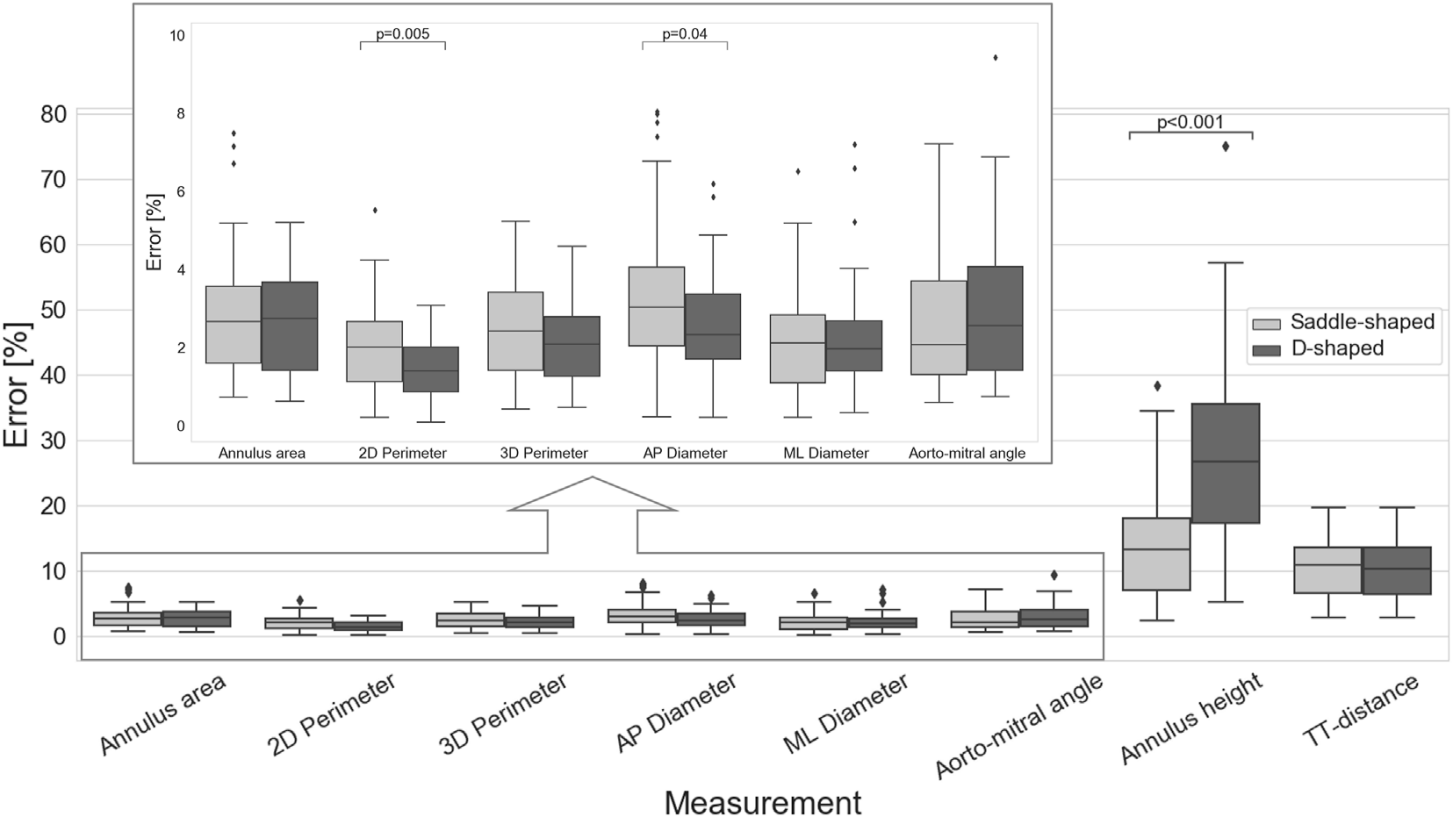
Inter-observer variability for the multiple measurements (*p*-values are shown for statistically significant differences between the saddle- and D-shaped annulus curves).

The impact of the discrepancy in the position of the landmarks on the mitral annulus measurements is represented as box plots in Figure 4. All relative errors were well below 10%, with the exception of the mitral annulus height and the inter-trigone distance. This trend in the inter-observer agreement was also seen for the intraclass correlation coefficients (ICC), with values between 0.23 and 0.71 for the annulus height and of 0.23 for the inter-trigone distance. In addition, the aorto-mitral angle was also associated with a low ICC, especially in the diastolic phase (ICC = 0.55 for saddle-shaped and ICC = 0.54 for D-shaped annulus), despite the low absolute and relative errors. These results might suggest that for these three measurements the average of the three observers cannot be reliably used as ground-truth for comparison with the automated approach. Significant differences were detected for the inter-observer agreement between the two annulus curve types for the 2D annulus perimeter (*p* = 0.005, the AP diameter (*p* = 0.04) and the annulus height (*p* < 0.001). The first two measurements were associated with a significantly lower error in the D-shaped annulus compared to the saddle shape, whereas the annulus height error was significantly higher in the D-shape configuration. A more detailed overview of the inter-observer variability for the saddle- and D-shaped annulus configurations can be found in Table 2, containing the mean absolute and relative inter-observer variability for the selected measurements, including the intraclass correlation coefficients values and corresponding 95% confidence interval.

**TABLE 2:** Inter-observer variability for mitral valve measurements.

	Saddle-shaped annulus					
	Diastole			Systole		
	Absolute	Relative	ICC (95%CI)	Absolute	Relative	ICC (95%CI)
Area 2D	0.27 ± 0.2 cm^2^	2.81 ± 1.8%	0.95 [0.89–0.98]	0.28 ± 0.2 cm^2^	2.91 ± 1.7%	0.93 [0.80–0.98]
Perimeter 2D	2.15 ± 1.2 mm	1.85 ± 1.0%	0.90 [0.74–0.96]	2.60 ± 1.5 mm	2.21 ± 1.4%	0.82 [0.50–0.93]
Perimeter 3D	2.76 ± 1.7 mm	2.22 ± 1.3%	0.86 [0.61–0.95]	3.43 ± 1.6 mm	2.78 ± 1.4%	0.77 [0.52–0.90]
AP Diameter	1.05 ± 0.6 mm	3.62 ± 1.9%	0.81 [0.61–0.91]	0.97 ± 0.6 mm	3.21 ± 2.2%	0.73 [0.52–0.87]
ML Diameter	0.62 ± 0.4 mm	1.57 ± 1.0%	0.93 [0.83–0.97]	1.08 ± 0.6 mm	2.84 ± 1.4%	0.83 [0.46–0.94]
Annulus height	0.99 ± 0.5 mm	18.2 ± 10.0%	0.38 [0.11–0.65]	0.71 ± 0.4 mm	11.1 ± 6.1%	0.71 [0.50–0.86]
Aorto-mitral angle	3.19 ± 2.4°	2.53 ± 2.0%	0.54 [0.29–0.76]	3.31 ± 1.7°	2.79 ± 1.5%	0.70 [0.48–0.85]
TT-distance	2.34 ± 1.0 mm	10.1 ± 4.7%	0.23 [0.00–0.51]	2.29 ± 1.0 mm	10.1 ± 4.7%	0.23 [0.00–0.51]
	Saddle-shaped annulus					
Area 2D	0.26 ± 0.17 cm^2^	2.67 ± 1.4%	0.95 [0.90–0.98]	0.26 ± 0.1 cm^2^	2.69 ± 1.5%	0.94 [0.86–0.98]
Perimeter 2D	1.69 ± 1.01 mm	1.46 ± 0.8%	0.94 [0.87–0.97]	1.68 ± 0.9 mm	1.46 ± 0.8%	0.92 [0.80–0.97]
Perimeter 3D	2.63 ± 1.51 mm	2.19 ± 1.2%	0.87 [0.76–0.94]	2.50 ± 1.2 mm	2.11 ± 1.1%	0.87 [0.73–0.94]
AP Diameter	0.66 ± 0.32 mm	2.33 ± 1.1%	0.90 [0.80–0.95]	0.87 ± 0.5 mm	2.96 ± 1.6%	0.81 [0.61–0.91]
ML Diameter	0.74 ± 0.40 mm	1.86 ± 0.9%	0.91 [0.75–0.96]	1.03 ± 0.7 mm	2.70 ± 1.8%	0.81 [0.46–0.93]
Annulus height	0.96 ± 0.59 mm	27.39 ± 16.4%	0.23 [0.00–0.52]	1.22 ± 0.7 mm	30.21 ± 14.4%	0.26 [0.01–0.54]
Aorto-mitral angle	3.48 ± 2.67°	2.74 ± 2.2%	0.55 [0.27–0.77]	3.83 ± 1.9°	3.18 ± 1.7%	0.67 [0.44–0.84]
TT-distance	2.34 ± 1.00 mm	10.1 ± 4.7%	0.23 [0.00–0.51]	2.29 ± 1.0 mm	10.1 ± 4.7%	0.23 [0.00–0.51]

AP, antero-posterior; CI, confidence interval; ML, medio-lateral; TT, trigone-to-trigone; ICC, intraclass correlation.

### 3.4 Automated mitral valve assessment

**FIGURE 5:**
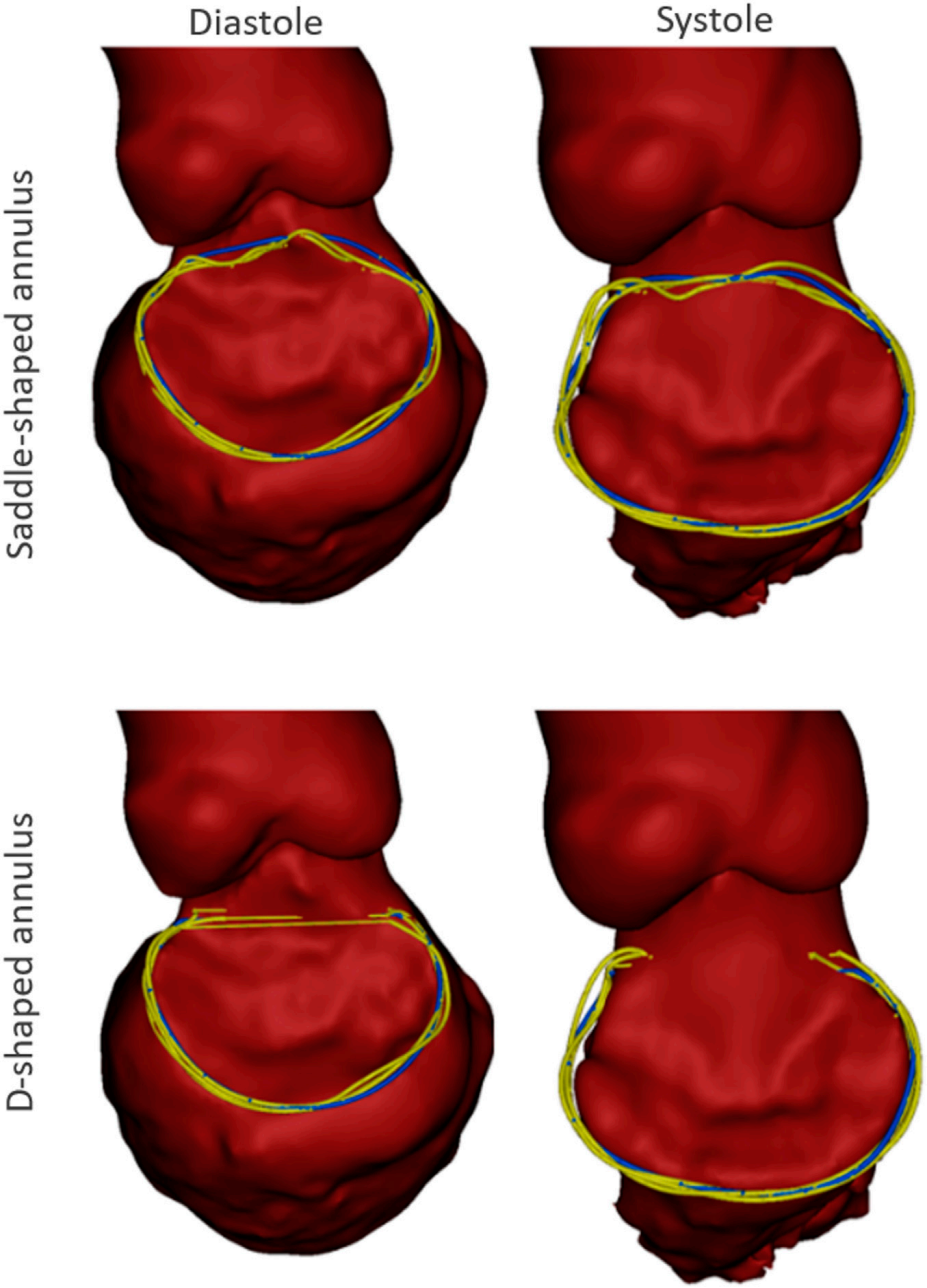
Example case with the manual annulus curves displayed in yellow and the automatically predicted annulus represented in blue, for both the saddle- (Top) and D-shaped (bottom) configurations, as well as for the diastolic (left) and systolic (right) phase. The LA was excluded for visualization purposes.

A representative example of the manually indicated and automatically predicted annulus curves is shown in Figure 5 for both cardiac phases and annulus configurations. In the top row, it is possible to appreciate how the manual and automated curves follow a similar path, with the main discrepancies occurring in the aorto-mitral curtain. In the bottom row, a smaller discrepancy is observed, although it is still possible to appreciate that the inter-trigone line is slightly different for the three manual indications.

A detailed inter-method variability analysis is included in Table 3. In the case of the saddle-shaped annulus, the relative errors for the annulus height and the inter-trigone distance were the highest, with values around 30%. Also, for the aorto-mitral angle, the inter-method analysis resulted in low ICC values, despite relatively low errors. These were the three measurements associated with the lowest interobserver agreement.

**TABLE 3:** Inter-method variability for mitral valve measurements.

	Saddle-shaped annulus					
	Diastole			Systole		
	Absolute	Relative	ICC (95%CI)	Absolute	Relative	ICC (95%CI)
2D Area	0.93 ± 0.7 cm^2^	10.34 ± 8.6%	0.90 [0.17–0.97]	0.95 ± 0.4 cm^2^	10.2 ± 5.9%	0.88 [0.10–0.97]
2D Perimeter	3.38 ± 2.8 mm	2.95 ± 2.5%	0.95 [0.88–0.98]	2.81 ± 2.0 mm	2.40 ± 1.7%	0.96 [0.90–0.98]
3D Perimeter	4.87 ± 2.8 mm	3.94 ± 2.2%	0.93 [0.82–0.97]	3.46 ± 2.5 mm	2.80 ± 2.0%	0.94 [0.86–0.98]
AP Diameter	5.65 ± 4.3 mm	12.01 ± 8.4%	0.71 [0.00–0.92]	3.59 ± 1.5 mm	11.9 ± 5.5%	0.61 [0.00–0.89]
ML Diameter	1.65 ± 1.5 mm	4.26 ± 3.8%	0.90 [0.76–0.96]	1.43 ± 0.9 mm	3.86 ± 2.4%	0.94 [0.85–0.98]
Annulus height	1.76 ± 1.1 mm	32.3 ± 21.7%	0.50 [0.00–0.79]	1.71 ± 1.2 mm	29.7 ± 26.6%	0.56 [0.00–0.83]
Aorto-mitral angle	3.58 ± 3.3°	2.78 ± 2.5%	0.82 [0.51–0.93]	5.27 ± 4.6°	4.42 ± 3.8%	0.69 [0.24–0.88]
TT-distance	6.71 ± 2.4 mm	29.1 ± 12.0%	0.21 [0.00–0.62]	6.16 ± 2.8 mm	28.1 ± 15.5%	0.17 [0.00–0.56]

	D-shaped annulus					
2D Area	0.66 ± 0.6 cm^2^	7.48 ± 6.8%	0.94 [0.77–0.98]	0.57 ± 0.4 cm^2^	5.94 ± 4.6%	0.95 [0.87–0.98]
2D Perimeter	3.14 ± 2.6 mm	2.82 ± 2.4%	0.96 [0.89–0.99]	2.38 ± 2.0 mm	2.11 ± 1.9%	0.97 [0.92–0.99]
3D Perimeter	3.30 ± 2.3 mm	2.78 ± 2.0%	0.96 [0.91–0.99]	2.85 ± 2.2 mm	2.40 ± 1.9%	0.96 [0.89–0.99]
AP Diameter	0.99 ± 0.8 mm	3.66 ± 3.4%	0.95 [0.88–0.98]	1.52 ± 1.10 mm	5.15 ± 4.7%	0.86 [0.64–0.94]
ML Diameter	1.63 ± 1.2 mm	4.13 ± 2.9%	0.92 [0.88–0.98]	1.28 ± 1.0 mm	3.42 ± 2.9%	0.94 [0.84–0.98]
Annulus height	1.96 ± 1.2 mm	52.8 ± 26.5%	0.26 [0.00–0.68]	1.58 ± 1.1 mm	36.7 ± 20.6%	0.51 [0.00–0.83]
Aorto-mitral angle	4.10 ± 3.5°	3.26 ± 2.9%	0.62 [0.42–0.80]	6.06 ± 4.6°	4.84 ± 2.1%	0.69 [0.15–0.88]
TT-distance	6.71 ± 2.4 mm	29.1 ± 12.0%	0.21 [0.00–0.62]	6.16 ± 2.8 mm	28.1 ± 15.5%	0.17 [0.00–0.56]

AP, antero-posterior; CI, confidence interval; ML, medio-lateral; TT, trigone-to-trigone; ICC, intraclass correlation.

**FIGURE 6:**
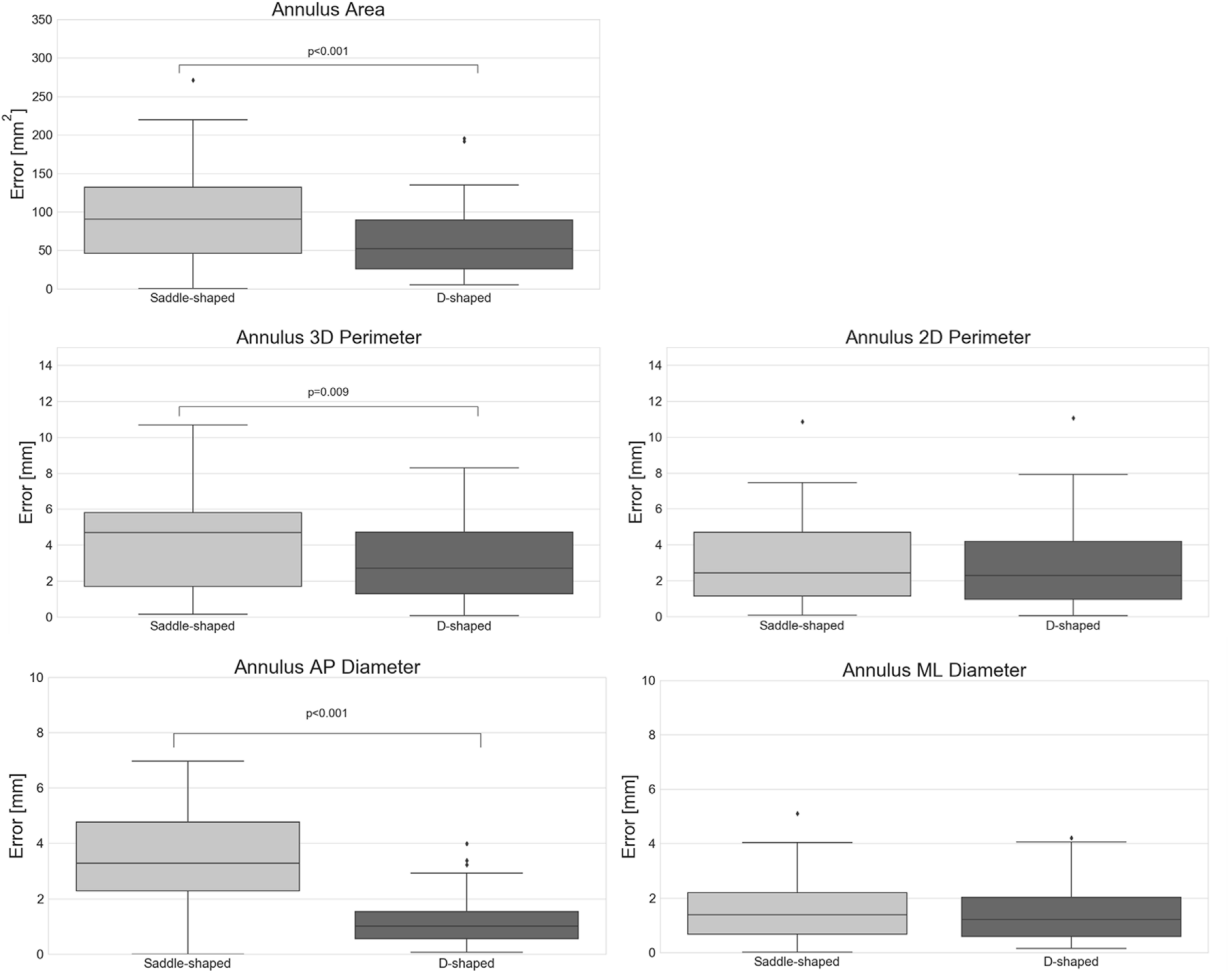
Absolute error of the automatically predicted annulus measurements for the saddle- and D-shaped annulus (*p*-values are shown for statistically significant differences between the saddle- and D-shaped annulus curves).

**FIGURE 7:**
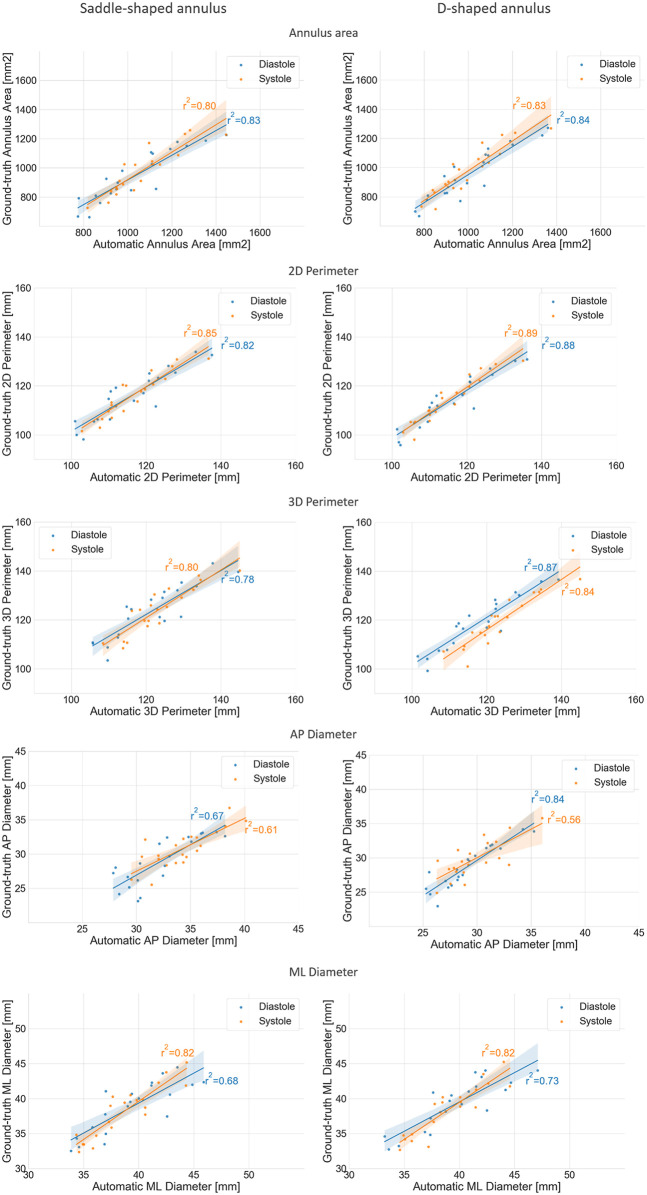
Regression plots comparing the mean annulus measurements obtained for the three observers and those calculated from the automatically detected landmarks, for both the saddle- (left) and D-shaped (right) configurations.

**FIGURE 8:**
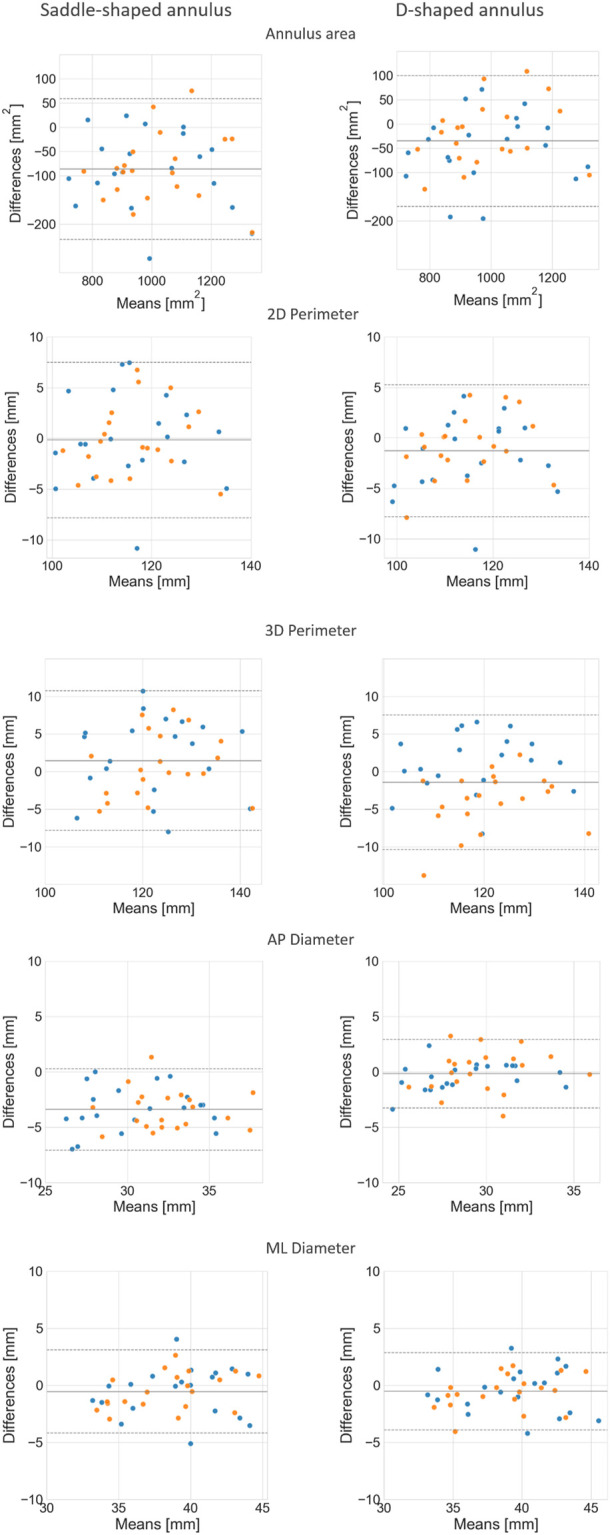
Bland-Altman plots comparing the mean annulus measurements obtained for the three observers and those calculated from the automatically detected landmarks, for both the saddle- (left) and D-shaped (right) configurations.

When considering the annulus area and antero-posterior diameter, both had a relative error of about 10%, a value considerably larger than those observed for the perimeters and medio-lateral diameter. The intraclass correlation coefficients show that these two measurements had limited reliability, with lower ICC values of 0.17 and 0.10 for diastole and systole, respectively, in the case of the projected area. For the AP diameter, the lower ICC values were of 0.00 in both phases. When analyzing the errors for the D-shaped annulus, it is possible to appreciate that these were considerably lower, and the ICC values higher when compared to the saddle-shaped representation. Figure 6 shows that the annulus area, 3D perimeter and AP-diameter are associated with statistically significant error differences between the two configurations, whereas the errors for the 2D perimeter and the ML-diameter remained nearly unchanged. For a more in-depth analysis of the effects of the annulus configuration in the different measurements, the linear regression and Bland-Altman plots can be consulted in Figure 7 and Figure 8, respectively.

The mean error for the mitral annulus area decreased from 94.3 ± 64.1 mm^2^ in the saddle-shaped annulus to 61.6 ± 38.6 mm^2^ in the D-shaped configuration. Furthermore, the coefficient of determination (*r* ^2^) increased for the diastolic and systolic phases. Also, the bias in the Bland-Altman analysis was reduced from almost −100 mm^2^ to about −50 mm^2^. The AP-diameter error decreased from 3.5 ± 1.77 mm for the saddle-shaped annulus to 1.25 ± 0.99 mm in the D-shaped configuration. Through the analysis of the Bland-Altman plot, it is possible to assess that the bias was eliminated, as its value decreased from about 4 mm to 0 mm. Regarding the coefficient of determination, while it largely increased from 0.67 to 0.84 for the diastolic phase, it decreased from 0.61 to 0.56 for the systolic phase.

There was a statistically significant difference in the relative and absolute errors of the 3D perimeter for the saddle- and D-shaped annulus. The mean absolute error decreased from about 4.2 ± 2.7 mm for the saddle-shaped annulus to 3.1 ± 2.2 mm for the D-shaped annulus. The linear regression also shows an improvement for the 3D perimeter in the D-shaped compared to the saddle-shaped configuration based on the slight increase in the coefficient of determination. In the Bland-Altman analysis, it is possible to appreciate that the bias shifted from 1.44 mm for the saddle-shaped annulus to -1.43 mm for the D-shaped curve. For the 2D perimeter, the bias increased from −0.17 for the saddle-shape to −1.29 for the D-shape. Lastly, the error for the medio-lateral diameter is almost the same for the two annulus configurations, around 1.5 ± 1.1 mm, and no obvious differences are observed between the two configurations in the regression and Bland-Altman analyses.

## 4 Discussion

Accurate pre-interventional assessment of the mitral valve is essential for successful outcome of TMVR procedures. This study evaluated the accuracy of a fully automated method for the detection of the main anatomical landmarks, which might contribute to a shorter and more consistent analysis process.

The inspection of the inter-method results indicates that the measurements generated using the proposed method showed good agreement with the measurements resulting from the user-driven approach, particularly for the diameters, perimeters, and area. Due to the low inter-observer agreement for the annulus height, aorto-mitral angle, and trigone-to-trigone distance, these measurements were considered ill-suited for the validation of the automatic method. The lower inter-observer agreement observed for these measurements is possibly related to the challenge of unambiguously defining the mitral annulus at the level of the aorto-mitral curtain. Because the fibrous skeleton is not associated with changes in image intensity compared to surrounding structures, discrepancies in the identification of the trigones position can occur, unavoidably leading to differences in the inter-trigone distance. Furthermore, the identification of the annulus horn is also prone to variations, contributing to large differences in the annulus height values. Because the aorto-mitral angle is directly related to the annulus plane orientation, variations in the annulus horn will also lead to significant differences in the angle with the aortic plane. The inter-method agreement was consistently higher for the D-shaped than for the saddle-shaped annulus. The fact that the relative error was significantly higher for the annulus area (*p* < 0.001), AP diameter (*p* < 0.001), and 3D perimeter (*p* = 0.009) in the saddle-shaped annulus compared to the D-shaped curve, and that no statistically significant differences were observed for the ML diameter and 2D perimeter, suggests that the discrepancies in the annulus curve might be local. The most logical explanation is that these errors occur in the anterior portion of the mitral annulus, as the delineation of the annulus in this region is known to be particularly challenging.

The understanding of the impact of these errors on the selected device as well as on the estimated device position is determinant to establish if the proposed automated method can be used in the pre-interventional planning of TMVR procedures. The device size is usually determined by a combination of measurements, namely area, perimeters, and diameters. This study suggested that by using the D-shaped configuration, the sizing of the device can be accurately performed. In case the saddle-shaped annulus description is used for the device selection, due to protocol specifications, a visual assessment and potential correction of the automatically generated annulus curve by an expert might be required. Regarding the position of the device in the cardiac anatomy, mainly determined by the annulus plane, the automated method estimated the mitral annulus and its center with a mean absolute difference of about 2.5 mm compared to the ground-truth. This is equivalent to the inter-observer variability, suggesting that an accurate planning can still be achieved with the proposed method. Nevertheless, further research is required to confirm this hypothesis.

This study presents inevitably some limitations. The number of validation cases is restricted, and a larger cohort might be required for a full understanding of the potential shortcomings associated to the method evaluated in this study. Another simplification related to the validation dataset is the fact that the subjects present no mitral valve pathology. It is expected that patients referred to TMVR procedures present multiple characteristics that might constitute a challenge in the automatic detection of the selected landmarks. Some examples are the presence of calcium, previous devices, challenging anatomy, such as extremely dilated heart chambers, among others. The evaluation of the proposed method on patients referred to TMVR procedures has been initiated and preliminary results for native cases indicate that the accurate identification of the mitral annulus is possible in patients presenting dilated left atrium or ascending aorta, as well as hypertrophic left ventricles. However, further validation is necessary to confirm these initial observations. An additional limitation is the fact that the present study evaluates the performance of the algorithm on two cardiac phases, whereas a thorough pre-interventional planning might require the analysis of the anatomy throughout the complete cardiac cycle. Finally, the automated algorithm is intended to be run on native cases, and therefore not suitable to plan valve-in-valve, valve-in-ring, and valve-in-MAC cases. These types of procedures represent a large part of the interventions currently being performed. However, due to the difficulty in establishing unambiguous guidelines for the device position on the existing structures, automating such procedures is currently implausible.

Despite these limitations, this study suggests that automated methods could contribute to increased consistency through the reduction of inter-observer variability, while shortening the time spent in the pre-interventional assessment.

## Data Availability

The data analyzed in this study is subject to the following licenses/restrictions: No authorization to share externally. Requests to access these datasets should be directed to PL, Patricia.Lopes@materialise.be.

## Abbreviations

AP, antero-posterior; ICC, intraclass correlation coefficient; LVOT, left ventricular outflow tract; ML, medio-lateral; MR, mitral regurgitation; SSM, statistical shape model; TMVR, transcatheter mitral valve replacement; TT, trigone-to-trigone.
